# Comparison of Cottle-Area-2 and Cottle-Area-3 in Computed Tomography Scans of Patients with Nasal Obstruction and Controls

**DOI:** 10.3390/diagnostics15111321

**Published:** 2025-05-24

**Authors:** Helen Heppt, Gerlig Widmann, Matthias Santer, Felix Riechelmann, Herbert Riechelmann, Aris I. Giotakis

**Affiliations:** 1 Department of Otorhinolaryngology, Medical University of Innsbruck, 6020 Innsbruck, Austria; helen.heppt@tirol-kliniken.at (H.H.); herbert.riechelmann@ikbnet.at (H.R.); arisgiotakis@gmail.com (A.I.G.); 2 Department of Radiology, Medical University of Innsbruck, 6020 Innsbruck, Austria; gerlig.widmann@i-med.ac.at; 3 Department of Orthopaedics and Traumatology, Medical University of Innsbruck, 6020 Innsbruck, Austria; felix.riechelmann@tirol-kliniken.at

**Keywords:** nasal patency, nasal obstruction, computed tomography, case-control studies, cross-sectional anatomy

## Abstract

**Background/Objectives**: Data that compare nasal Cottle-area-2 (i.e., nasal valve) and Cottle-area-3 are sparce. We intended to compare these areas in subjects with and without nasal obstruction. **Methods**: We compared cross-sectional areas, derived by computed tomography, of Cottle-area-2 (CT-CSA_COT-2_) and Cottle-area-3 (CT-CSA_COT-3_), in cases planned for surgery due to chronic nasal obstruction and controls with trauma not involving the head. In these cases, we investigated the correlation of the size of narrow and wide sides with active anterior rhinomanometry (AAR). **Results**: In 56 cases, CT-CSA_COT-2_ were 15% smaller than CT-CSA_COT-3_ (all *p* < 0.007). However, both were similarly large in 56 controls (all *p* > 0.2). Both narrow sides of the CT-CSA_COT-2_ and CT-CSA_COT-3_ were significantly smaller in cases (69 ± 23 mm^2^ and 79 ± 28 mm^2^, respectively) than in controls (91 ± 21 mm^2^; *p* < 0.001 and 93 ± 21 mm^2^; *p* = 0.004, respectively). However, only the size of the total nasal airway of CT-CSA_COT-2_ was significantly smaller in cases (*p* < 0.001), not that of CT-CSA_COT-3_ (*p* > 0.2). Correlations of AAR with CT were significant only on the narrow sides (all *p* < 0.037), but not on the wide sides (all *p* > 0.2). **Conclusions**: In contrast to Cottle-area-3, the total nasal airway of Cottle-area-2, i.e., nasal valve, was smaller in patients with nasal obstruction, the latter of which may not be easily identified before nasal surgical procedures.

## 1. Introduction

Otorhinolaryngologists have traditionally used the Cottle classification to categorize the areas of the nasal cavity [1,2]. Areas 1, 2, 3, 4 and 5 indicate the nostril, the nasal valve, the area underneath the cartilaginous and bony vault, the anterior aspects of the nasal cavity, including the heads of the turbinates and the infundibulum, and the posterior aspect of the nasal cavity, including the tails of the turbinates, respectively [2].

Usually, otorhinolaryngologists mention Cottle areas when they refer to nasal surgical procedures. Cottle himself has published multiple surgical papers along with Loring [3,4,5]. Thorough attempts to objectively assess the Cottle areas have been made by acoustic rhinometry and active anterior rhinomanometry (AAR) [6,7]. However, despite their significant advantages, functional rhinometric procedures have several disadvantages. They are error-prone, examiner dependent, not easily available and not verifiable [8].

On the contrary, computed tomography (CT) is less error-prone, not examiner dependent, easily available and verifiable. A further main advantage of CT is the availability of hospital-based controls [9,10], which is not the case with functional rhinometric procedures.

Recent attempts using this advantage revealed interesting results. These were significant asymmetry of the nasal floor [10] and narrower cross-sectional areas anterior to the piriform aperture [9], but not posterior to it [11], in patients with nasal obstruction compared to control patients. A further advantage of these studies was the standardized measurement of cross-sectional areas by using easily-found bony landmarks [9,11].

With this study, we aimed to use CT scans to compare cross-sectional areas (CT-CSA) of Cottle-area-2 (i.e., nasal valve) and Cottle-area-3 between patients with nasal obstruction and controls. In patients with nasal obstruction, we also investigated the correlation between CT-CSA of Cottle-area-2 and Cottle-area-3, and AAR. The latter was examined separately on the narrow and wide nasal side to investigate a potential pathophysiologic effect.

## 2. Materials and Methods

### 2.1. Study Design

This was a hospital-based retrospective study. Eligible cases were adult subjects with a preoperative cone beam CT-scan, undergoing septo(-rhino)plasty due to chronic nasal obstruction, from January 2017 to December 2020, at the University Department of Otorhinolaryngology, Head and Neck Surgery. We applied the SPSS function random sample to these cases to generate a gender-balanced sample. Adult subjects who attended the Department of Orthopedics and Traumatology from January 2017 to December 2020 for management of trauma not involving the head and face served as controls. Here, the routine workup included a multi-slice CT of the whole body. Exclusion criteria included opacification of the nasal cavity or sinuses, dysmorphic syndromes of the head and face, or trauma of the facial bones. In cases where AAR with Otopront Rhino-Sys system (Otopront, Hohenstein, Germany) was available, we investigated the relationship of AAR with CT. Since CT-scans and AAR were not systematically performed on the same day, we used only values before decongestion. The local ethics committee approved the study protocol on 12 December 2019 (1261/2019).

### 2.2. CT-Planes

CT-CSA were assessed manually by an individual investigator. The investigator measured separately the right and left nose in mm^2^ [9]. The software Syngo-share-view (Siemens Healthcare Diagnostics GmbH, Vienna, Austria) was used to visualize DICOM data sets of cone beam CT and multi-slice CT [9,10,11], the accuracy of which is considered similar due to no significant differences of the target registration error [12].

**Figure 1:**
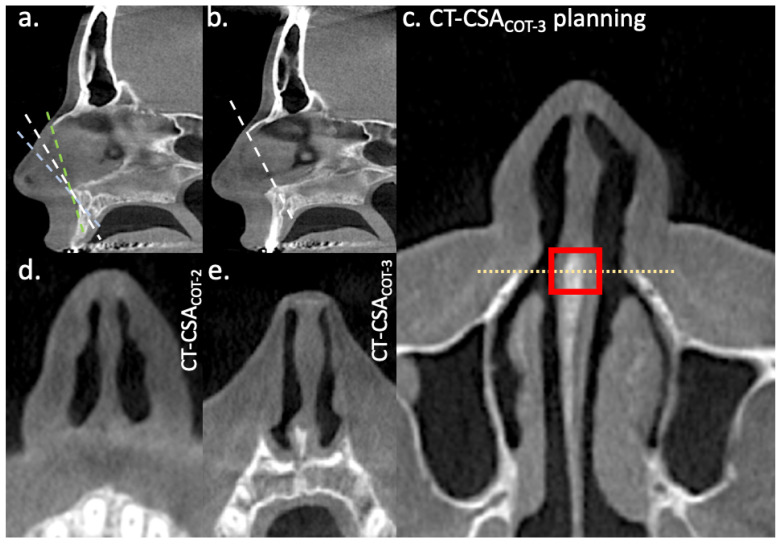
Identification of the planes of Cottle-area-2 and Cottle-area-3 at the sagittal plane of the septum. The selected oblique planes for (**a**) Cottle-area-2 and (**b**) Cottle-area-3 are indicated by the white dashed line. The blue and green dashed line in (**a**) indicates the oblique planes of the CT-CSA titled about 30° and 60° to the nasal floor, respectively, examined in another study [9]. The cross-sectional areas of (**d**) CT-CSA_COT-2_ and (**e**) CT-CSA_COT-3_ correspond to (**a**) and (**b**), respectively. The head of the inferior turbinate is visible in the plane of Cottle-area-3 (**e**). Planning of the CT-CSA_COT-3_ is depicted in (**c**). The yellow pointed line indicates the level of the anterior borders of the ascending processes of the maxilla, and the red rectangle indicates where the plane of Cottle-area-3 starts.

The plane of Cottle-area-2 was defined by two points. The first one was the anterior nasal spine. The second one was the middle of a line drawn along the nasal dorsum that started from the anterior edge of the intranasal suture (K-area) and ended when it intersected the line of the CT-plane titled 30° to the nasal floor that was examined in a similar study (Figure 1) [9]. The plane of Cottle-area-2 was tilted about 45° to the nasal floor (CT-CSA_COT-2_).

The plane of Cottle-area-3 was defined by two points. The first was the anterior border of the intranasal suture. The second was the most cranial edge of the premaxilla at the level of the anterior borders of the ascending processes of the maxilla (CT-CSA_COT-3_; Figure 1). The latter was found by scrolling the axial CT scan from cranially to caudally, where a point was set at the first encountered premaxilla structure at the level of the anterior borders of the ascending processes of the maxilla (Figure 1).

### 2.3. Data Analysis

We used the SPSS 26.0 statistic package (SPSS Inc., Chicago, IL, USA) to analyze the data. We used tables for count data, and means, standard deviations and 95% confidence intervals (CI) for metric data. We used the Shapiro–Wilk test to check for normality of distribution of variables. Correlations for continuous parameters were examined with Pearson’s correlation coefficient. Correlations were categorized as strong, if r > |0.8|, moderate, if |0.8| > r > |0.6| and weak, if r < |0.6|. The following three parameters were derived from the raw data.

The first parameter was the narrow cross-sectional area. Assuming that nasal obstruction is more depended on the narrow sides, we compared the CT-CSA of Cottle-area-2 and Cottle-area-3 on the narrow side between cases and controls, and for correlation with AAR. The variables of AAR were displayed for the left and right nose. To compare them to the narrow and wide nasal sides of CT, AAR variables were re-assigned to narrow and wide nasal sides based on the narrow and wide nasal sides per plane in CT.

The second parameter was the bilateral cross-sectional area. Here, we added the CT-CSA of both sides for Cottle-area-2 and Cottle-area-3, separately. The bilateral cross-sectional areas represented a measure of the internal width of the nose [9] in Cottle-area-2 and Cottle-area-3. Bilateral cross-sectional areas were used for comparison between cases and controls, and for correlation with AAR.

The third parameter was the ratio of the narrow to bilateral cross-sectional area. Here, we examined the ratio of the narrow- to the bilateral CT-CSA in the planes of Cottle-area-2 (CT-CSA_COT-2-ratio_) and Cottle-area-3 (CT-CSA_COT-3-ratio_), separately. These ratios indicated the asymmetry of the internal nose’s width of each CT-CSA. These ratios were compared between cases and controls.

## 3. Results

### 3.1. Study Population

During the study period, 1005 patients underwent a nasal surgical procedure. From these patients, we drew a random sample of 60 subjects, which was gender-balanced. Of these 60 subjects, 56 subjects were eligible and included. Thirty-one were men. The median age was 31 years (range: 18–60 years). The Department of Orthopedics and Traumatology and of Radiology provided a control sample, which was equally sized to the cases (56 trauma controls) and with balanced gender distribution (30 men). In the controls, the median age was 27 years (lower to upper quartile: 20.25 to 41 years), and in the cases, it was 31 years (24.25 to 48 years; Mann–Whitney U test; *p* = 0.071).

### 3.2. CT-CSA of Cottle-Area-2 and Cottle-Area-3

CT-CSA of both Cottle-area-2 and Cottle-area-3 were normally distributed (Shapiro–Wilk test; *p* > 0.096). CT-CSA of Cottle-area-2 ranged from 17 to 191 mm^2^ (mean value: 93 mm^2^), and CT-CSA of Cottle-area-3 ranged from 34 to 188 mm^2^ (mean value: 102 mm^2^). Both CT-CSA of Cottle-area-2 and Cottle-area-3 were similar between men and women (independent-samples T-test; both *p* > 0.2).

In the cases, CT-CSA of Cottle-area-2 were significantly smaller than CT-CSA of Cottle-area-3 (paired samples T-test; all *p* < 0.007 in right, left, narrow, wide and total noses). On the contrary, when compared within controls, CT-CSA of Cottle-area-2 were neither larger nor smaller than CT-CSA of Cottle-area-3 (paired samples T-test; all *p* > 0.2 for right, left, narrow, wide and total noses; Table 1).

### 3.3. Narrow Cross-Sectional Areas

**Figure 2:**
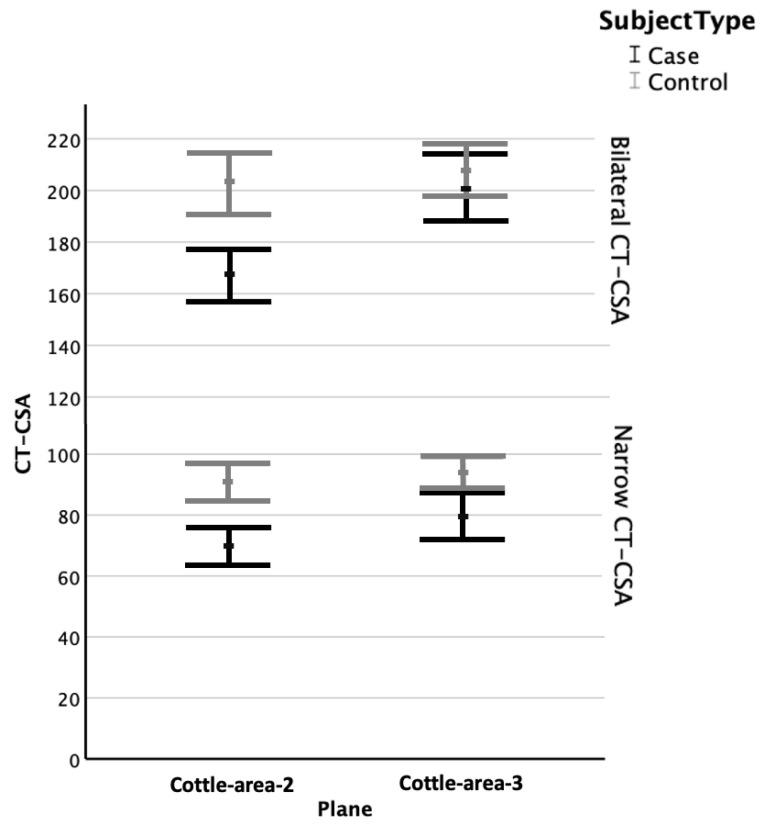
Comparison of nasal cross-sectional areas in CT (CT-CSA) of subjects with nasal obstruction (cases; black) and trauma controls (grey). X-Axis: Plane. Y-Axis: Size of CT-CSA in mm^2^; mean values (points) with error bars indicating lower and upper bound of 95% confidence intervals. Note significant differences between cases and controls at the plane of Cottle-area-2 (left) for bilateral (upper) and narrow (lower) CT-CSA (both *p* adjusted for age <0.001). Note significant differences between cases and controls at the plane of Cottle-area-3 (right) for narrow (lower) CT-CSA (*p* = 0.004), but not for bilateral (upper) CT-CSA (*p* > 0.2).

CT-CSA of Cottle-area-2 on the narrow side correlated significantly with age (r = 0.29; *p* = 0.002). Adjusted for age, these were significantly smaller in the cases (69 ± 23 mm^2^) than in the controls (91 ± 21 mm^2^; *p* < 0.001; Figure 2).

CT-CSA of Cottle-area-3 on the narrow side did not correlate significantly with age (*p* > 0.2). These were significantly smaller in cases (79 ± 28 mm^2^) than in controls (93 ± 21 mm^2^; independent-samples T-test; *p* = 0.004; Figure 2).

### 3.4. Bilateral Cross-Sectional Areas

Bilateral CT-CSA of Cottle-area-2 correlated significantly with age (r = 0.33; *p* < 0.001). Adjusted for age, bilateral CT-CSA of Cottle-area-2 were significantly smaller in cases (167 ± 37 mm^2^) than they were in controls (203 ± 44 mm^2^; *p* < 0.001; Figure 2).

Bilateral CT-CSA of Cottle-area-3 did not correlate with age (*p* > 0.2). In contrast to the bilateral CT-CSA of Cottle-area-2, bilateral CT-CSA of Cottle-area-3 did not differ between cases (201 ± 48 mm^2^) and controls (208 ± 39 mm^2^; independent-samples T-test; *p* > 0.2; Figure 2).

### 3.5. Ratio of Narrow Cross-Sectional Areas to Bilateral Cross-Sectional Areas

CT-CSA_COT-2-ratio_ and CT-CSA_COT-3-ratio_ were not normally distributed (both *p* < 0.001). Both variables did not correlate significantly with age (both *p* > 0.2).

**Figure 3:**
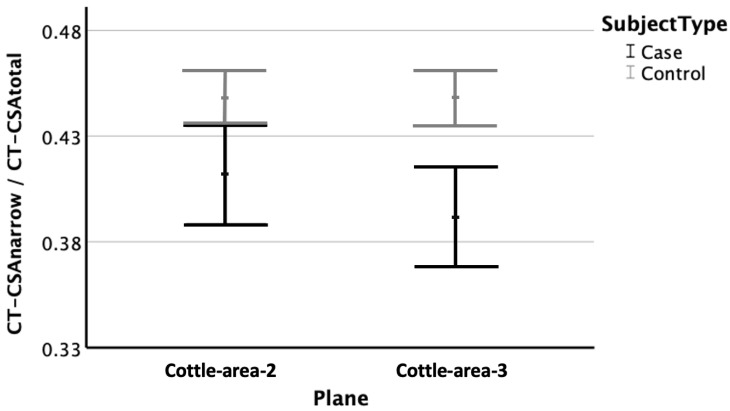
Comparison of nasal asymmetry between subjects with nasal obstruction (cases; black) and trauma controls (grey). X-Axis: Plane. Y-Axis: CT-CSA ratio, i.e., the narrow cross-sectional area of the nose divided by the total airway area of the nose; mean values (points) with error bars indicating lower and upper bound of 95% confidence intervals. Note significant differences between cases and controls in the plane of Cottle-area-2 (left; *p* = 0.015). Note also larger significant differences in the plane of Cottle-area-3 (right; *p* < 0.001), indicating the more frequent encountered nasal asymmetry at the plane of Cottle-area-3 in subjects with nasal obstruction.

CT-CSA_COT-2-ratio_ were significantly smaller in the cases (median: 44%; lower quartiles 39% to upper quartiles 47%) than they were in the controls (46%; 42% to 49%; Mann–Whitney U test; *p* = 0.015; Figure 3). Similarly, CT-CSA_COT-3-ratio_ were significantly smaller in the cases (41%; 33% to 47%) than they were in the controls (47%; 42% to 48%; Mann–Whitney U test; *p* < 0.001; Figure 3).

### 3.6. Correlation of CT-CSA with AAR

**Figure 4:**
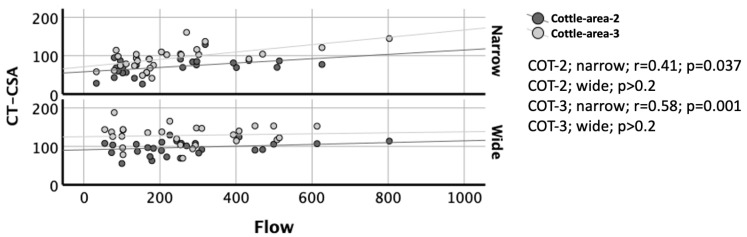
Correlation of CT-CSA in the planes of Cottle-area-2 and Cottle-area-3 with flow of active anterior rhinomanometry. Y-Axis: CT-CSA in mm^2^. X-Axis: Flow in mL/s. Dark grey and light grey circles, as well as dark grey and light grey lines indicate the Cottle-area-2 (COT-2) and Cottle-area-3 (COT-3), respectively. The upper and lower diagram indicate the narrow and wide nasal side, respectively. Note the significant correlation (r = 0.58) between the CT-CSA of Cottle-area-3 and flow on the narrow nasal sides (*p* = 0.001) but not on the wide nasal sides (*p* > 0.2).

**Figure 5:**
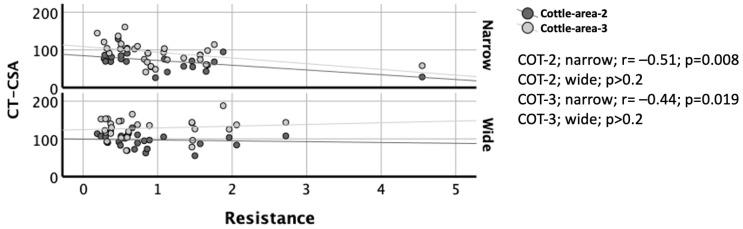
Correlation of CT-CSA in the planes of Cottle-area-2 and Cottle-area-3 with resistance of active anterior rhinomanometry. Y-Axis: CT-CSA in mm^2^. X-Axis: Resistance in sPa/mL. Dark grey and light grey circles, as well as dark grey and light grey lines indicate the Cottle-area-2 (COT-2) and Cottle-area-3 (COT-3), respectively. The upper and lower diagram indicate the narrow and wide nasal side, respectively. Note the significant negative correlation (r = −0.56) between the CT-CSA of Cottle-area-2 and resistance on the narrow nasal sides (*p* = 0.008) but not on the wide nasal sides (*p* > 0.2).

Complete AAR data were available in 26 subjects. When examined pathophysiologically based on the narrow and wide nasal sides, correlations between AAR and CT were significant only on the narrow sides of the nose (all *p* < 0.037) but not on the wide sides (all *p* > 0.2; Figure 4 and Figure 5). On the narrow sides, correlations between AAR and CT were weak. As expected, flow and resistance were smaller and larger, respectively, on the narrow sides than on the wide sides (Table 2).

## 4. Discussion

Cottle classification is even today a matter of debate [13]. Still, data about the comparison of Cottle areas between patients with and without nasal obstruction are scarce. Recent studies have allowed the comparison of nasal anatomy in CT-scans, between subjects with nasal obstruction and patients without it, using a hospital-based setting [9,10,11]. We intended to take advantage of this setting to compare Cottle-area-2 and Cottle-area-3 between subjects with and without nasal obstruction. We chose to investigate Cottle-area-2 since Cottle assigned it as “nasal valve” (i.e., internal nasal valve), and we chose to investigate Cottle-area-3 due to its close proximity to Cottle-area-2.

Our results revealed that the CT-CSA of both Cottle-areas were significantly smaller on the narrow sides in the subject group with nasal obstruction. However, only the total nasal area of Cottle-area-2 (i.e., nasal valve), but not of Cottle-area-3, was significantly smaller in the cases (Figure 2). These results indicate that both Cottle-area-2 and Cottle-area-3 contribute to nasal obstruction. These results may further imply that two important parameters should be considered during septoplasty or septorhinoplasty. When addressing Cottle-area-2 (i.e., nasal valve), one may consider widening the total nasal airway at this plane and not only focusing on straightening the nasal septum. On the contrary, when addressing Cottle-area-3, it could be sufficient to correct the asymmetry of the nasal cavities, e.g., by straightening the nasal septum, without necessarily widening the total nasal airway at this plane. Interventional studies are required to further investigate these findings.

Nevertheless, the results of this study do not support a greater impact of Cottle-area-2 over Cottle-area-3 on nasal obstruction. One additional investigated parameter was the ratio of the size of the narrow CT-CSA to the size of both CT-CSA per each plane, which was a measure of the asymmetry of the nasal airway. The results revealed that the ratio of Cottle-area-3 was much more significant (*p* < 0.001) for nasal obstruction than the ratio of Cottle-area-2 (*p* = 0.015; Figure 3). This actually implied that the smallest nasal ratio (narrow side to the total nasal airway) in subjects with nasal obstruction was more frequently encountered in Cottle-area-3 than in Cottle-area-2.

Previous studies have also demonstrated the importance of the nasal valve in nasal obstruction. Moche and coauthors measured the nasal valve area in subjects with obstructed and unobstructed internal nasal valves. They reported significant smaller nasal valves in obstructed patients (38 mm^2^) than in unobstructed patients (51 mm^2^). The nasal valves of the mentioned study were smaller than those in the current study. However, the assessment was performed in the axial plane [14]. Poetker and coauthors highlighted the importance of the assessment of the nasal valve at the proper plane. The authors examined the nasal valve angle at the traditional coronal plane and the nasal base view plane. The latter was much similar to the perpendicular orientation to the nasal airflow used for the nasal valves in the current study. They found that angles were more consistent with the classic description (10° to 15°) at the nasal base view plane than they were at the traditional coronal plane (8°) [15].

The current study is one of the few case-control naval valve studies found in the literature. This hospital-based setting, i.e., subjects with nasal obstruction and trauma controls, has been successfully used in several studies [9,10,11]. This study was novel in many ways. It included 112 subjects, many more than similar studies [14,15,16,17,18,19]. Furthermore, the current study examined simultaneously Cottle-area-2 and Cottle-area-3, in subjects with nasal obstruction and controls. Cottle-area-2 (i.e., nasal valve) was significantly smaller than Cottle-area-3 in cases, which is in line with the common knowledge that the nasal valve is the narrowest part of the airway. Interestingly, in contrast to Cottle-area-3, cross-sectional areas located just posterior to it, were not able to predict nasal obstruction [11]. This indicates the importance of Cottle-area-3.

NOSE score was not available in this study. Nevertheless, active anterior rhinomanometry was performed in subjects with nasal obstruction. AAR data were available only in 26/56 subjects, since the data were partly collected from the time period during the restrictions of COVID-19. AAR is a standardized functional technique that evaluates nasal patency [20]. It measures the inspiratory airflow and resistance. Here, cross-sectional areas of the nose were compared to AAR in novel ways. Firstly, we used standardized, reproducible CT-CSA of Cottle-area-2 and Cottle-area-3. Furthermore, instead of the usual right and left noses, narrow and wide noses were assessed.

These allowed for a significant observation. Correlations of CT with AAR were significant only on the narrow sides of the nose, but not on the wide sides (Figure 4 and Figure 5). This may indicate that flow and resistance variables are more dependent on the size of a cross-sectional area when it is smaller than a critical area, and they are less dependent on the size of a cross-sectional when it is larger than a critical area. This has been partly described by Garcia and coauthors. The authors proposed that the nasal resistance is described by the Bernoulli obstruction theory, which predicts a strong correlation between resistance and cross-sectional area but only for severe constrictions, where the cross-sectional area is smaller than a critical area [21].

The design of Cottle-area-2 and Cottle-area-3 in CT might seem complicated. However, the design of the CT-CSA_COT-3_ intended to be representative of the Cottle-area-3 three-dimensional space and aimed for easiness in measuring, reproducibility, and standardization. In the classic schematic of Cottle [2], the apex of Cottle-area-3 triangle is located slightly posterior to the anterior nasal spine. For this reason, we chose the bony landmark to be the most cranial part of the premaxilla at the level of the anterior borders of the ascending processes of the maxilla as the apex of the triangle. Furthermore, the plane of Cottle-area-2 was approximately in the middle of two nearby CT-CSA (titled 30° and 60° to the nasal floor in a similar study) [9]. This design had significant advantages and overcame certain difficulties, resulting in a clinically significant CT-CSA. Nevertheless, despite the accuracy of the design, it was not completely based on bony landmarks, and this is considered a limitation of the study.

A further limitation of the study was the absence of a NOSE score. The documentation of a NOSE score at our department was not a routine practice during the study period. Moreover, controls were somewhat younger than patients surgically treated for nasal obstruction (*p* = 0.071). Therefore, all comparisons of cases and controls were adjusted for age. Furthermore, the definition of the narrow and wide areas of the nose per plane based on the CT might have been complicated. However, it offered interesting insights to the pathophysiology of the nose.

The absence of a NOSE score is a limitation due to several more reasons. The obvious one was the lack of correlation of Cottle-area-2 and Cottle-area-3 to a subjective assessment of nasal patency. However, there were more implications to consider. The feeling of breathing through the nose may be affected by multiple more anatomical sites anterior, posterior or even between Cottle-area-2 and Cottle-area-3. However, the latter might be covered by previous studies to some extent [9,11,22]. Most importantly, mucosal factors (e.g., allergies or irritants), psychological factors or even the trigeminal system contribute to an unknown extent to the feeling of breathing through the nose. The lack of a NOSE score did not allow us to investigate the effects of these factors compared to that of Cottle-area 2 and Cottle-area 3. Lastly, the presence of a NOSE score or another subjective assessment method of nasal patency might allow us to investigate to which extent Cottle-area 2 and Cottle-area 3 can predict the feeling of breathing through the nose.

One additional point of criticism might involve the assignment of trauma subjects to the control group. Here, one advantage was the availability of a CT scan as part of a routine workup. Furthermore, nasal obstruction does not generally alter the risk of trauma. Therefore, we considered these subjects to be suitable as a control group. However, the absence of a NOSE score and other subjective or objective assessment methods of nasal patency did not allow us to exclude with certainty that some trauma subjects did not suffer from nasal obstruction. This was also considered a limitation of this study design.

The size of our study sample was chosen empirically based on similar studies [9,11], which revealed significant differences with a similar sample size. We considered 55 subjects per group as an adequate number to draw a significant result from with an independent-samples t-test (α = 0.05), a moderate effect size (d = 0.54) and a power of 0.8.

Future studies could compare preoperative and postoperative NOSE scores to investigate which nasal area (e.g., Cottle-areas or others) and to what extent it contributes more to a better postoperative NOSE score. Other factors, such as mucosal condition (e.g., allergies or irritants), psychological factors or even the trigeminal system should be documented in order to neutralize confounding factors.

## 5. Conclusions

In contrast to Cottle-area-3, the total nasal airway area of Cottle-area-2 (i.e., nasal valve) was smaller in patients with nasal obstruction, the latter of which may not be easily identified before nasal surgical procedures.

## Figures and Tables

**Table 1:** CT-CSA of Cottle-area-2 and Cottle-area-3.

Plane ^1^	Side	Cases	Controls
Cottle-area-2	Right	83 ± 27 (25–130)	103 ± 25 (64–191)
	Left	83 ± 26 (17–143)	102 ± 27 (59–170)
	Narrow	69 ± 23 (17–130)	91 ± 21 (49–143)
	Wide	97 ± 22 (55–143)	112 ± 27 (65–191)
	Total	167 ± 37 (98–272)	203 ± 44 (129–296)
Cottle-area-3	Right	99 ± 35 (41–166)	104 ± 24 (56–159)
	Left	102 ± 37 (34–188)	106 ± 25 (50–159)
	Narrow	79 ± 28 (34–161)	93 ± 21 (50–140)
	Wide	122 ± 31 (60–188)	115 ± 24 (69–159)
	Total	201 ± 48 (105–322)	208 ± 39 (128–300)

^1^ In mm^2^ (mean value ± standard deviation; minimum–maximum).

**Table 2:** Inspiratory flow and resistance of Cottle-area-2 and Cottle-area-3 based on narrow or wide nasal side.

Plane	AAR ^1^ Variable	Narrow Side ^2^	Wide Side ^2^
Cottle-area-2	Flow (mL/s)	235 ± 169 (33–626)	264 ± 173 (55–803)
	Resistance (sPa/mL)	1.05 ± 0.89 (0.28–4.55)	0.86 ± 0.61 (0.19–2.72)
Cottle-area-3	Flow (ml/s)	233 ± 172 (33–803)	270 ± 163 (55–613)
	Resistance (sPa/mL)	1.01 ± 0.83 (0.19–4.55)	0.88 ± 0.69 (0.24–2.72)

^1^ Active anterior rhinomanometry before decongestion. ^2^ (mean value ± standard deviation; minimum–maximum).

## Data Availability

Data used in this study can be requested by the corresponding author upon reasonable request. The data are not publicly available due to privacy restrictions.

## Abbreviations

The following abbreviations are used in this manuscript:

CT	Computed tomography
AAR	Active anterior rhinomanometry
CT-CSA	Computed tomography cross-sectional areas
COT-2	Cottle-area-2
COT-3	Cottle-area-3
COT-2-ratio	Cottle-area-2 ratio
COT-3-ratio	Cottle-area-3 ratio
CI	Confidence intervals
SD	Standard deviation
